# Comparison of the mechanical characteristics of produced nanofibers by electrospinning process based on different collectors

**DOI:** 10.1016/j.heliyon.2023.e23841

**Published:** 2023-12-17

**Authors:** Sajjad Sheikhi, Aazam Ghassemi, S. Mohammad Sajadi, Mohammad Hashemian

**Affiliations:** a Department of Mechanical Engineering, Najafabad Branch, Islamic Azad University, Najafabad, Iran; b Department of Nutrition, Cihan University-Erbil, Kurdistan Region, Iraq; c Department of Mechanical Engineering, Khomeinishahr Branch, Islamic Azad University, Khomeinishahr, Iran

**Keywords:** Nanofiber, Electrospinning, Collector, Polyacrylonitrile, Dimethylformamide, Mechanical property

## Abstract

Polymer nanofiber in nanofibrous membrane produced by electrospinning process can be employed in various fields such as medical engineering, environmental engineering, biotechnology, energy, tissue scaffolds, and protective clothing. In these applications, the mechanical properties of the nanofibrous membrane should be studied to get long-life durability. In the current study, nanofibers are obtained from electrospinning of polyacrylonitrile (PAN) solution in Dimethylformamide (DFM) solvent. Nanofibers are produced with disc, cylinder, wire drum, parallel bars and polygon collectors and their mechanical properties are examined and compared. For this study, a tensile testing machine with special jaws was applied. According to the Scanning Electron Microscope (SEM) images, the average diameter of the produced nanofibers ranges from 300 to 340 nm. In addition, nanofiber layers have a thickness of 0.03 mm. They were cut in the 10 × 25 mm^2^ size; then, the tensile test was performed. Results show that produced nanofiber layers by rotating cylinder collector have the highest ultimate strength while the disk collector results in the highest Young's modulus in produced samples.

## 1 Introduction

In the last few decades, electrospinning has been considered an efficient method for the production of nanofibers, and the simplicity and ease of the electrospinning method have led to many creativities and innovations in its initial process. Previous researches have shown that electrospinning can produce various organic, ceramic, and fiber composite materials with controllable diameters. In addition, electrospinning has been developed for the direct production of nanofibers with a hollow core-shell structure. Scientists have found in their research that it is necessary to study the relationship between the secondary structure of electrospun nanofibers and process parameters. Research into the production of electrospun nanofiber secondary structures has introduced new methods for designing advanced electrodes, catalyst sources, and sensor devices. Especially hollow nanofibers with a circular cross-section are ideal channels for the passage of nanofluids. In general, research in electrospinning has led to the application of nanofibers in a wide range of fields [[1], [2], [3], [4], [5]].

Electrospinning is fast developing from a single-fluid process [6,7] to coaxial [8], tri-axial [9], side-by-side [10], and other complicated processes [11]. Correspondingly, uniaxial [12], core-shell [13], Janus [14], tri-layer core-shell nanostructures [15] have been reported for a wide variety of functional applications. However, one of the most important properties of mechanical performance is often ignored in literature, which is vital for functional applications regardless of the complexity [16].

Doshi and Reneker [17] invented the preparation of polyethylene oxide fibers electrically. In this process, after dropping the polymer solution drop and until the electric field overcomes the surface tension, a charged jet exits the solution to the collector, and fibers are formed in the range of nanometer diameter. Inai et al. [18] used a table-mounted folding plate to collect separate nanofibers. Conductive plates were installed near a paper support tape to collect the fibers regularly. In this case, the separate nanofibers were collected on a paper strip in the desired arrangement. Except for the nanofibers located in the strip center, the excess nano-fibers were separated from it. Then, the sample prepared in the tensile strength test at the nanoscale was measured. Ohgo et al. [19], Zong et al. [20], Huang et al. [21,22]and Huang et al. [23], Li et al. [24], and Pedicini and Farris [25] researched mechanical properties of the nanofibers with random arrangement collected by an aluminum plate. Also, Bhattarai et al. [26], Lee et al. [27,28], Wnek et al. [29], Khil et al. [30], Nagapudi et al. [31], and Ding et al. [32] investigated mechanical properties of regularly arranged nanofiber laminates produced by rotary collectors. Katti et al. [33] investigated the effect of parameters such as needle diameter, polymer solution concentration and voltage per unit length on the morphology and diameter of electrospun nanofibers. In their study, antibiotics were loaded into a polylactic glycolic polymer solution (PLGA) to design a drug delivery system and wound healing. In general, they showed that glycolic polylactic nanofibers could be brought to the desired diameter through changes in proportional processing parameters, and antibiotics such as cefazolin can be added to the nanofibers. Therefore, glycolic polylactic nanofibers have shown their potential as antibiotic delivery systems in wound healing. Hong et al. [34] produced antimicrobial Polyvinyl Alcohol (PVA) nanofibers containing silver nitrate nanoparticles with the chemical formula of AgNO_3_. According to their observations, if a silver nitrate polymer solution is used in polyvinyl alcohol with a weight percent of 10 to 0.1, the electrospinning process can be successful. During their research on the surface of nanofiber structures, Yoo et al. [35] found that electrospun nanofibers with a huge area-to-volume ratio are very suitable because of their potential applications for medical devices, tissue engineering scaffolds, and drug delivery carriers. Kizildag et al. [36] investigated conductive polyaniline nano-fibers (PANi) in polyvinyl alcohol (PVA/PANi) produced by electrospinning with rotary collectors and found that conductive nanofibers can be used for a wide range of applications such as electromagnetic interference protection, antistatic applications, gas sensors, tissue engineering scaffolding, biomedical applications, nanoelectronic devices, etc. Also, they produced conductive nanofibers (PVA/PANi) via electrospinning successfully. Wang et al. reported fabrication of electrospun composite fibers based on Poly vinylidene fluoride (PVDF) and multi-walled carbon nanotubes (MWCNTs) [37]. They found an increase in mechanical and electrical properties of fibers after incorporating MWCNTs into the PVDF fibers. Sun et al. [38] investigated the applications of electrospun nanofibers in energy. These nano-fibers can be widely used in energy storage systems due to very high surface-to-volume ratio and porosity of electrospun nanofibers. They focused mainly on using nano-fibers in energy storage devices, for example, lithium batteries, fuel cells, dye-sensitized solar cells, and supercapacitors. In another study, Itoh et al. [39] investigated the morphology, and mechanical properties of PVA nano-fibers spun by free-surface electrospinning. Their research showed that due to the electrical nature of electrospinning, the electrical and ionic conductivity of the polymer solution plays an essential role in this process and the morphology of the fibers. Utilizing three different collectors, Polycaprolactone electrospun fibers have been produced by De Prá et al. [40]. Based on the results, rotational speed and electrostatic forces are dominant phenomena in stretching fibers collected with rotating drum and static collector, respectively.

According to the authors’ knowledge, there is no comparison between the mechanical properties and geometry of nanofibers obtained by electrospinning of 13 % PAN solution in DMF solvent produced by various fixed and rotary collectors. So, this issue is investigated in the present study. First, five types of disk, cylinder, wire drum, parallel bars, and polygon collectors were designed and made, two of which are rotary, and three are fixed models. Then, after the electrospinning process, the mechanical properties of the produced fibers by five collectors are compared.

## 2 Materials and methods

**Fig. 1:**
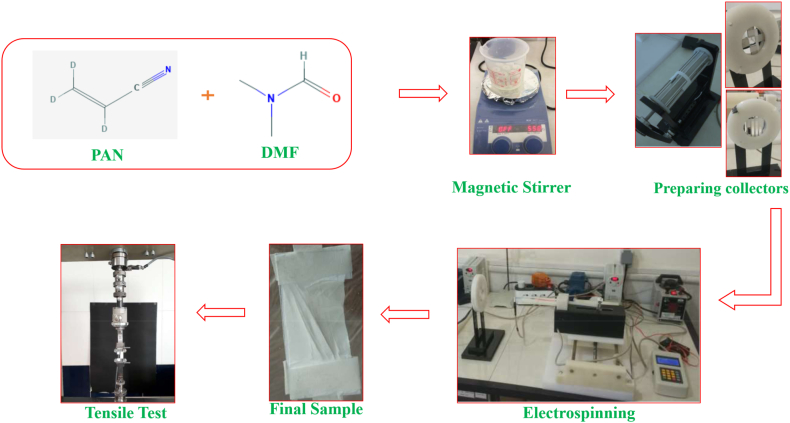
Schematic of making a sample of nanofibers by electrospinning process and its mechanical characterization.

Fig. 1 shows a schematic of the parts preparation and the electrospinning process. These cases are explained in more detail below.

### 2.1 Modeling of collectors

**Fig. 2:**
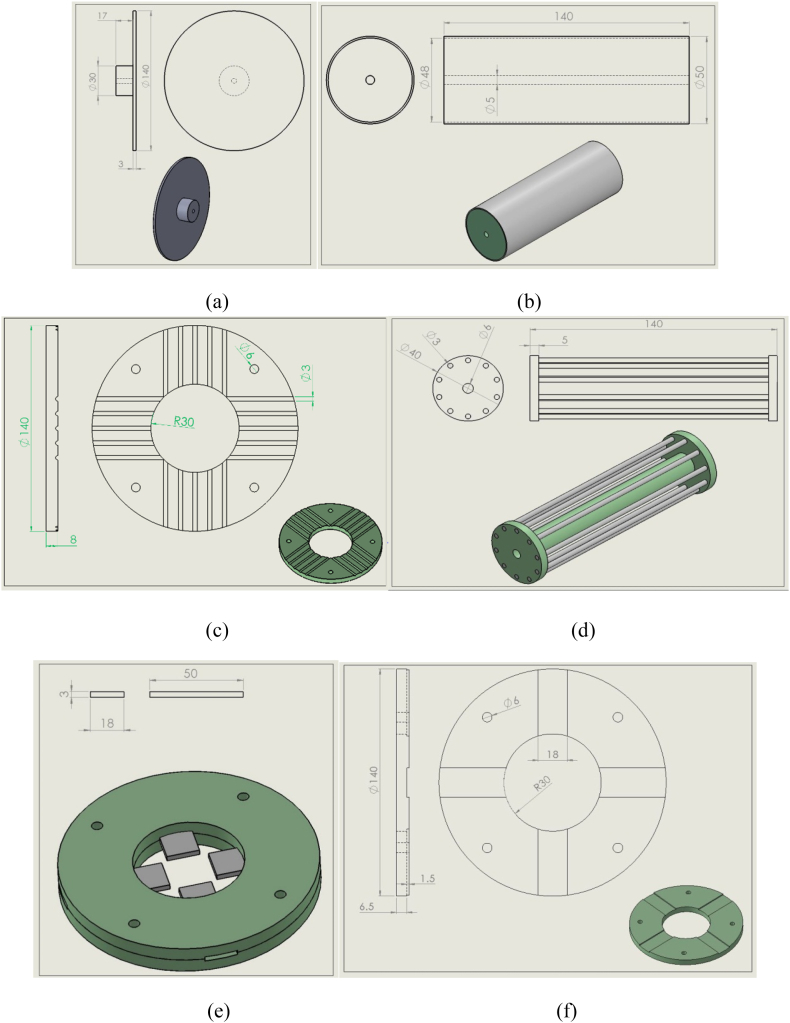
Modeled (a) plate or disc; (b) cylinder; (c) parallel bars; (d) wire drum; (e,f) polygon collectors.

Solidwork software was employed to model the collectors. The collectors were designed to be as small as possible compared to other collectors and reduce their manufacturing costs without losing efficiency. Fig. 2 shows the modeled collectors in this research along with their related construction drawings (Fig. 2. (a) plate or disc; (b) cylinder; (c) parallel bars; (d) wire drum; (e,f) polygon collectors). The dimensions mentioned in the pictures are in millimeters. It should be noted that the three disks, parallel bars, and polygon collectors are fixed, and the cylinder and wire drum collectors are rotary.

### 2.2 Manufacturing of collectors

**Fig. 3:**
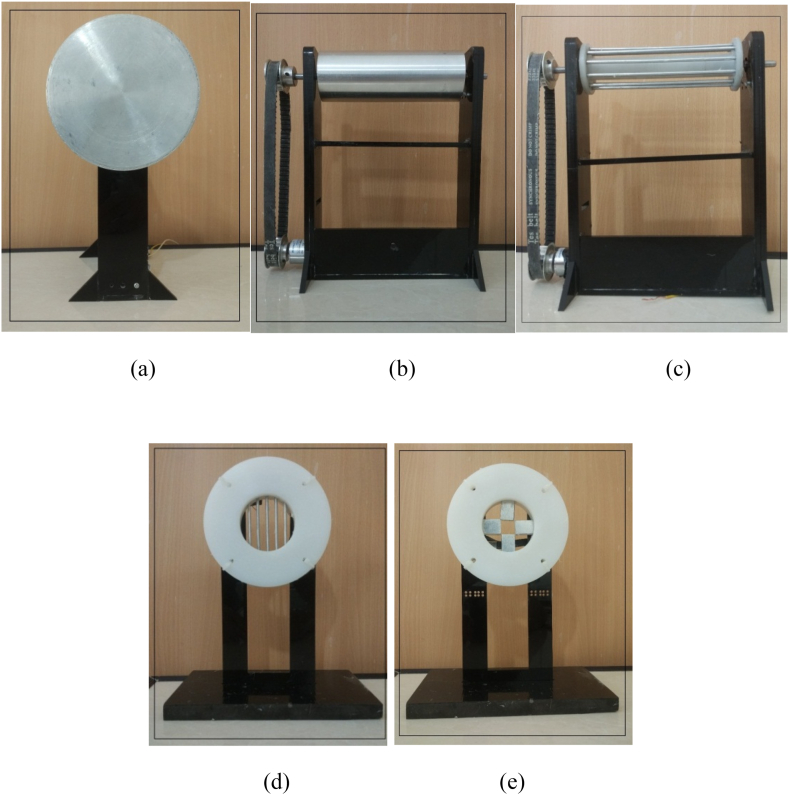
Manufactured (a) plate or disc; (b) cylinder; (c) wire drum; (d) parallel bars; (e) polygon collectors.

The main body of the collectors on which the fibers are collected is made of aluminum, which has good conductivity. The rest of the collector components are made of polyethylene, which is non-conductive. The cylinder collector was made of an aluminum tube mounted on a round piece of polyethylene. Also, in wire drum and parallel bars collectors, only the bars, and in the polygon collector, only the four sides are made of aluminum. In the construction of collectors, ordinary lathe and milling machines and equipment such as column drills, taps, reamers, etc., are used. The bases are made of plexiglass and carbon dioxide (CO_2_) laser cutting machines. Fig. 3 illustrates the manufactured collectors (Fig. 3. (a) plate or disc; (b) cylinder; (c) wire drum; (d) parallel bars; (e) polygon collectors).

### 2.3 Electrospinning

**Fig. 4:**
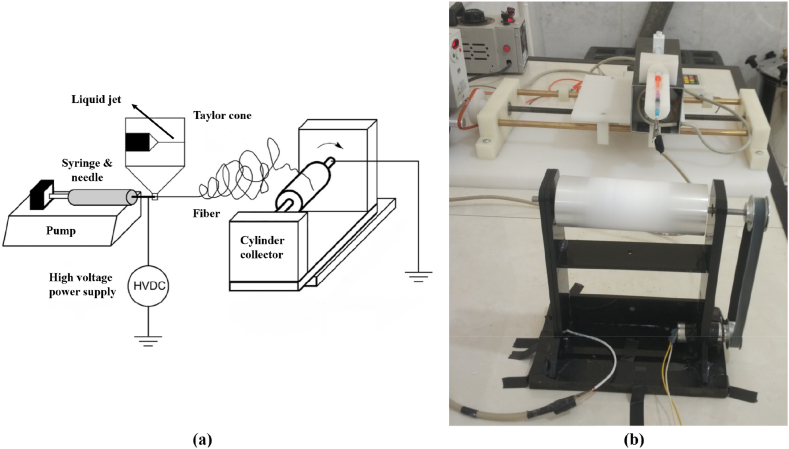
Electrospinning process with cylinder collector; (a) Schematic diagram; (b) Manufactured setup.

Fig. 4 shows a schematic diagram of the electrospinning process with a cylinder collector (Fig. 4a), and the manufactured setup in this research (Fig. 4b).

In this study, PAN solution in DFM solvent was used to perform the electrospinning process. The concentration of the solution is 13 %. To achieve this concentration, 1.3 gr of PAN powder must be dissolved in 10 ccs of DFM. The electrospinning process conditions are the same for all collectors because the fibers are made with the same production conditions and compared in terms of different properties. The distance from the tip of the needle to the collector is 15 cm, the duration of electrospinning is 15 min, the system voltage is 150 V and the feed rate is 0.6 ml per hour. The rotational speed of rotary collectors is 1980 rpm. It should be noted that the electrospinning process is performed in laboratory conditions at a temperature of 23 °C.

### 2.4 Tensile test

**Fig. 5:**
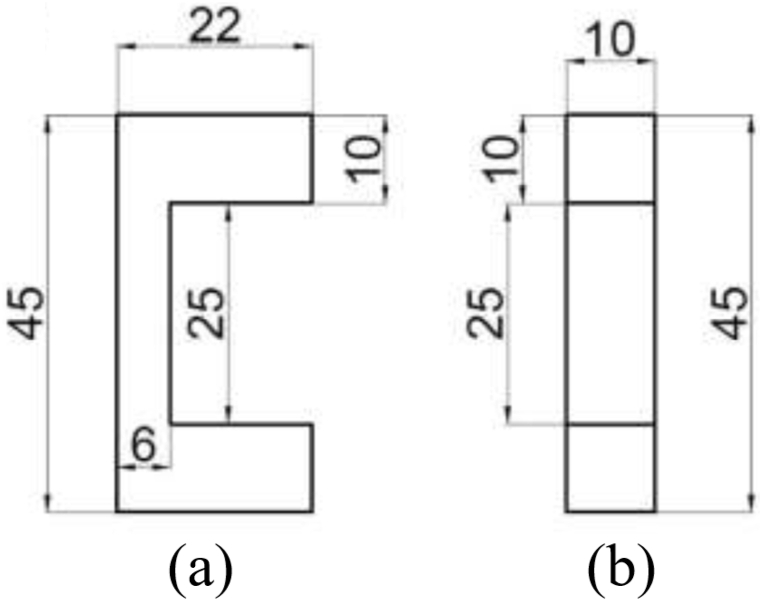
Nanofiber layer retaining mold.

According to DIN EN ISO 2062, the tensile test specimens must first be collected on the mold shown in Fig. 5 a and Fig. 5b and then cut with a very sharp blade measuring 10 × 25 mm^2^ and placed in the jaws for the tensile test. It should be noted that the thickness of the fiber layer is 0.03 mm.

**Fig. 6:**
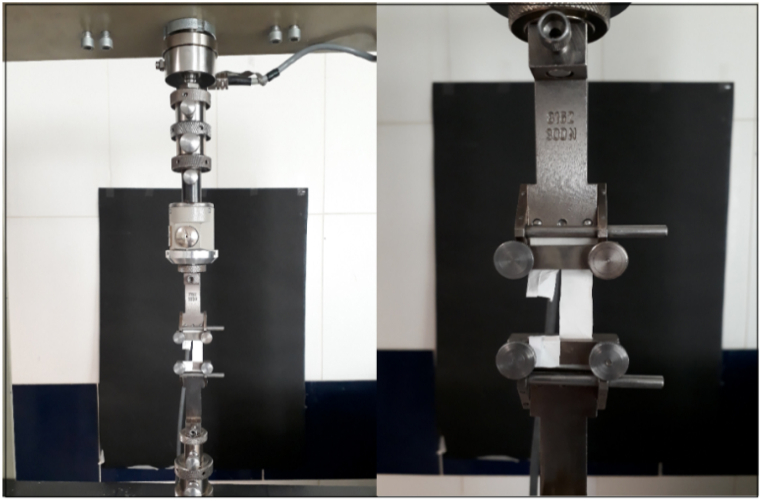
Jaws holding the fiber layer in the tensile test device.

The main problem in preparing tensile test specimens is to separate the nanofiber layers from the collector because these layers, due to being charged, collect quickly after being separated from the collector surface and cannot be installed on the tensile test mold. To solve this problem, the molds are glued to the surface of the collectors using paper glue, and after the electrospinning operation, the mold is separated from the collector surface with a layer of fibers. Fig. 6 shows the jaws of the tensile test device and how the nanofiber layer is placed. Moreover, the placement of the fiber layers is very important and should be the same in terms of shape and orientation, and the speed of the tensile test is 10 mm/min.

The method of preparing the tensile test sample is that first, the nanofiber layer with the relevant mold is placed between the jaws, then the paper part is cut, and the machine is ready to work. These steps are repeated for each tensile test. In this study, three tensile tests were performed for each collector, ending the tensile test with 15 repetitions. The reason for performing repetitions is the possibility of reliability of the tensile test data.

## 3 Results and discussion

**Fig. 7:**
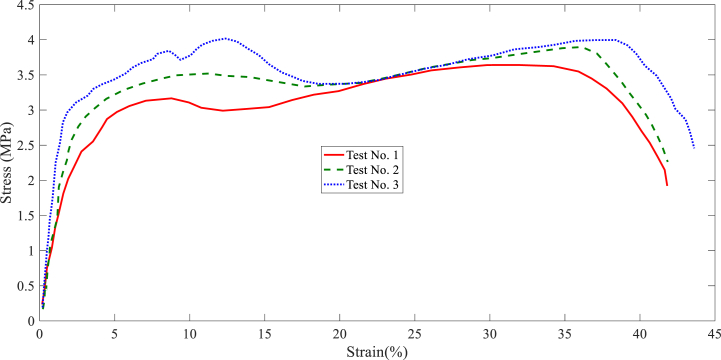
Tensile test of the nanofibers layers manufactured by disc collector.

**Fig. 8:**
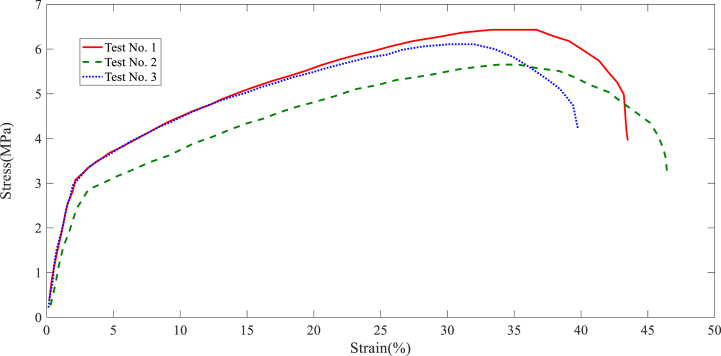
Tensile test of the nanofibers layers manufactured by cylinder collector.

**Fig. 9:**
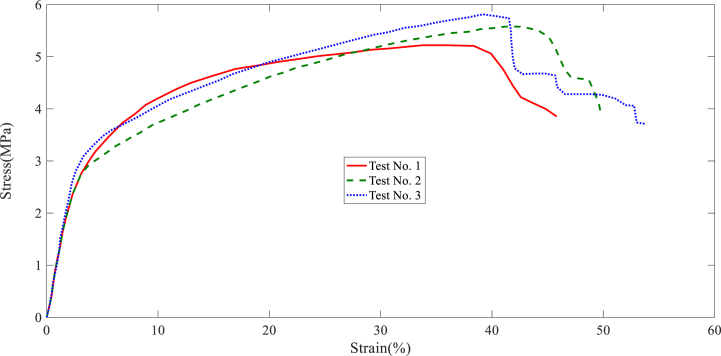
Tensile test of the nanofibers layers manufactured by wire drum collector.

**Fig. 10:**
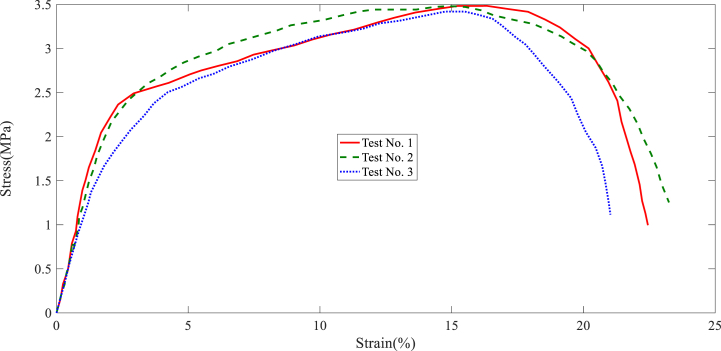
Tensile test of the nanofibers layers manufactured by parallel bars collector.

**Fig. 11:**
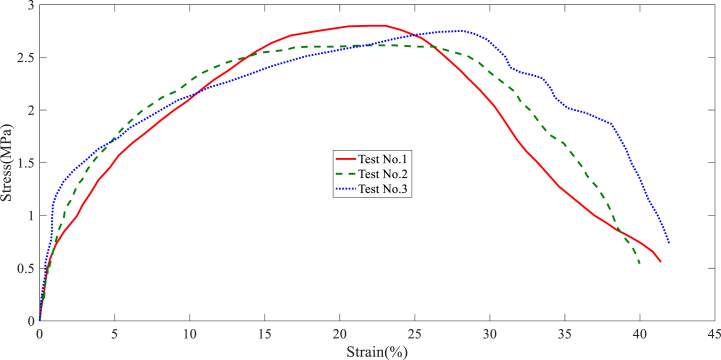
Tensile test of the nanofibers layers manufactured by polygon collector.

In this section, the results of the layer tensile test are stated. The tensile test results of each collector are shown in Fig. 7, Fig. 8, Fig. 9, Fig. 10, Fig. 11. It is worth noting that the information provided is related to the three replications of the tensile test. The red, green, and blue graphs show the first, second, and third iterations of the samples obtained from different collectors in the following diagrams.

Based on the above figures, the mechanical properties of the samples obtained from the electrospinning process with different collectors can be summarized in Table 1.

**Table 1:** Results related to the repetitions of tensile test in nanofiber layers obtained from different collectors.

Collector Type	*Test No.*	$F_{\max}$	Elongation at $F_{\max}$	Ultimate Stress	Strain at maximum stress	E-Modulus
		kN	mm	MPa	%	MPa
Disc	1	106.398	8.64	3.641	31.984	101.39
	2	110.894	5.89	3.897	36.138	122.3
	3	121.684	13.05	3.996	38.395	116.85
	Average	112.992	9.19	3.845	35.506	113.51
Wire drum	1	162.251	5.32	5.220	33.764	51.87
	2	151.492	7.59	5.577	41.161	63.55
	3	171.208	9.91	5.813	39.186	65.35
	Average	161.65	7.6	5.537	38.037	60.25
cylinder	1	189.864	8.82	6.432	36.654	83.47
	2	167.356	8.57	5.652	33.854	62.96
	3	185.856	8.36	6.107	30.141	73.59
	Average	181.02	8.58	6.064	33.550	73.34
Parallel bars	1	104.235	3.58	3.484	16.348	83.1
	2	106.107	3.69	3.479	14.740	70.39
	3	99.348	2.89	3.420	14.752	65.57
	Average	103.23	3.38	3.461	15.280	73.02
Polygon	1	84.672	5.56	2.800	21.956	62.81
	2	79.453	4.89	2.614	21.693	73.59
	3	81.738	5.79	2.751	28.197	64.19
	Average	81.95	5.41	2.722	23.949	66.86

**Fig. 12:**
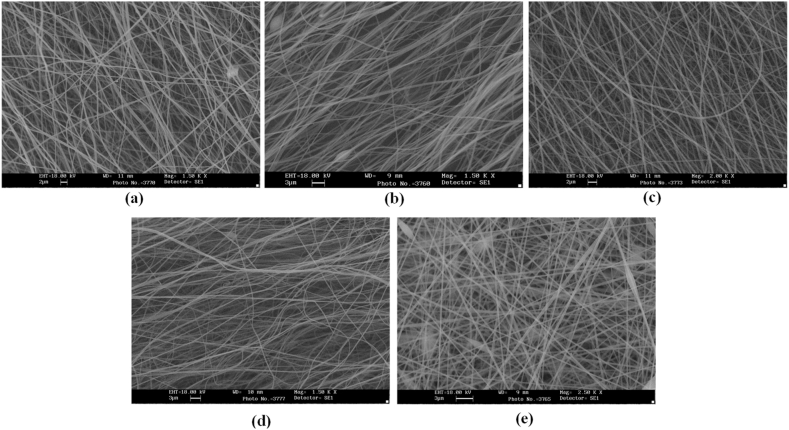
SEM of the specimen manufactured by (a) disk collector, (b) wire drum collector, (c) cylinder collector, (d) parallel bars collector, (e) polygon collector.

According to Table 1, the highest ultimate stress of the produced nanofiber layers is related to the cylinder collector; 6.064MPa (see average ultimate stress values). In addition, the models made with wire drum, disc, parallel bars, and polygon collectors are in the following ranks in terms of ultimate stress. In terms of Young's modulus, the highest value refers to specimens made with disc collectors; 113.51MPa. In this regard, manufactured specimens with cylinder, parallel bars, polygon, and wire drum collectors are in the next ranks. An SEM can be used to examine the diameter of a polymer nanofiber [17]. Scanning electron microscopy images of the disc (plate), wire drum, cylinder, parallel bars, and polygon collectors are shown in Fig. 12 (Fig. 12a disk collector, (b) wire drum collector, (c) cylinder collector, (d) parallel bars collector, (e) polygon collector).

By examining this figure, it was identified that the average diameter of nanofibers is between 300 and 340 nm. Also, the fibers in the disk collector are collected irregularly. However, in-cylinder, wire drum, and parallel bars collectors, the fibers tend to be parallel-arranged, with the wire drum collector having the most parallel fiber arrangement. In the case of polygon collectors, the fibers tend to be 90° closer to each other. As the pictures illustrate, the conductive metal arrangement of the collectors is directly related to the fibers' arrangement. By changing the arrangements, the fibers' orientation changes because of the stretching of the fibers towards the conductive metal.

## 4 Conclusion

In this research, nano-fibers were obtained by electrospinning of 13 % PAN solution in DMF solvent, manufactured in the form of a nanofiber layer with the disc (plate), cylinder, wire drum, parallel bars, and polygon collectors, were evaluated and compared in terms of their mechanical properties. According to the results of the tensile test, the cylinder rotary collector has higher ultimate stress than other collectors. However, in terms of Young's modulus, it makes a fixed disc collector of nano-fibers with the highest Young's modulus. According to the electron microscope images, the wire drum collector can create the best parallel arrangement.

## Data availability

All data to support the conclusions have been either provided or are otherwise publicly available.

## CRediT authorship contribution statement

**Sajjad Sheikhi:** Data curation, Conceptualization. **Aazam Ghassemi:** Software, Methodology, Investigation. **S. Mohammad Sajadi:** Writing – review & editing, Software. **Mohammad Hashemian:** Validation, Resources, Investigation.

## Declaration of competing interest

The authors declare that they have no known competing financial interests or personal relationships that could have appeared to influence the work reported in this paper.
